# Anti-Inflammatory and Barrier-Stabilising Effects of Myrrh, Coffee Charcoal and Chamomile Flower Extract in a Co-Culture Cell Model of the Intestinal Mucosa

**DOI:** 10.3390/biom10071033

**Published:** 2020-07-11

**Authors:** Laura Weber, Katrin Kuck, Guido Jürgenliemk, Jörg Heilmann, Bartosz Lipowicz, Cica Vissiennon

**Affiliations:** 1 Institute of Medical Physics and Biophysics, Medical Faculty, Leipzig University, Härtelstraße 16-18, 04107 Leipzig, Germany; 2 Repha GmbH Biologische Arzneimittel, Alt-Godshorn 87, 30855 Langenhagen, Germany; bartosz.lipowicz@repha.de; 3 Pharmaceutical Biology, Institute of Pharmacy, University of Regensburg, Universitätsstraße 31, 93053 Regensburg, Germany; katrin.kuck@chemie.uni-regensburg.de (K.K.); guido.juergenliemk@chemie.uni-regensburg.de (G.J.); joerg.heilmann@chemie.uni-regensburg.de (J.H.)

**Keywords:** myrrh, coffee charcoal, chamomile flower, IBD, intestinal barrier, inflammation, mucosa, co-culture cell model

## Abstract

Recent clinical evidence suggests the efficacy of a traditional herbal medicinal product containing myrrh (*Commiphora molmol* Engl.), coffee charcoal (*Coffea arabica* L.) and chamomile flower dry extract (*Matricaria chamomilla* L.) in the therapy of inflammatory bowel diseases (IBD). However, the mechanisms of action in this context have not been entirely elucidated. The present study aimed to evaluate the effects of myrrh, coffee charcoal and chamomile flower extract on the inflammatory cross talk between immune and intestinal epithelial cells together with the resulting intestinal barrier disorders. A complex co-culture cell model consisting of intestinal epithelial cell (IEC) monolayers (Caco-2, HT29-MTX-E12) and macrophages (THP-1) was established for the simultaneous investigation of these two IBD characteristics. The lipopolysaccharide (LPS) activation of the macrophages led to a pro-inflammatory mediator release and thereby an inflammatory stimulation of IECs with chemokine release and reduced barrier function. The effects of the individual plant extracts and a ternary combination on inflammatory mediator release (IL-6, TNF, IL-8, MCP-1, PGE2) was quantified by ELISA. The transepithelial electrical resistance (TEER) of IEC monolayers was measured to evaluate the effects on the barrier function. Budesonide served as a positive control. All three plant extracts exhibited anti-inflammatory properties via the inhibition of the inflammatory mediator release to a varying extent. An intestinal barrier stabilising effect was observed for myrrh and coffee charcoal. Myrrh exerted the most distinct pharmacological activity. Dose reducing and synergistic interactions emerged within the threefold combination. Thus, our results provide a mechanistic basis for the use of the herbal combination of myrrh, coffee charcoal and chamomile flower extract in IBD treatment and underline the potential benefits of the phytotherapeutic multi-component/multi-target approach in this complex pathogenesis.

## 1. Introduction

Inflammatory bowel diseases (IBD), with the main forms Crohn’s disease and ulcerative colitis, affect a large and growing proportion of patients worldwide, especially in developed and newly industrialising countries, and constitute a severe and life-long burden [1]. Though the aetiology and pathogenesis remain to be fully unravelled, the current understanding is that the interaction of genetic, environmental and microbial factors leads to imbalances in intestinal homoeostasis resulting in an inadequate immune response as well as intestinal barrier impairment [2,3,4]. Beyond IBD, the dysfunction of the intestinal barrier is also associated with various other diseases e.g., coeliac disease and irritable bowel syndrome, which highlights the importance of a functional mucosal barrier [5]. 

IBD proceed with a chronically relapsing course, which implies particularly high requirements for the efficacy and safety of the treatment. Therapeutic approaches aim to control the symptoms, increase the quality of life and prevent disease progression and complications [2]. Studies report that a large proportion of IBD patients show interest in complementary and alternative medicine (CAM) as a therapeutic approach, with herbal medicinal products being among the most commonly used options [6,7]. Scarce evidence on CAM in IBD treatment currently limits the acceptance amongst health care providers and complicates its integration into clinical practice. Further scientific validation of CAM approaches can help to utilize their full potential in IBD therapy and allow for a holistic risk benefit assessment in collaboration between the physician and patient [6,8].

Recent clinical findings suggest that a herbal combination of myrrh (*Commiphora molmol* Engl.), coffee charcoal (*Coffea arabica* L.) and chamomile flower dry extract (*Matricaria chamomilla* L.), known as Myrrhinil-Intest^®^, is effective in the maintenance therapy of patients with ulcerative colitis [9]. The traditional herbal medicinal product is used in the treatment of gastrointestinal disorders in Germany. It was recently incorporated into the Updated S3-Guideline Colitis ulcerosa by the German Society for Digestive and Metabolic Diseases [10]. Pharmacological in vitro studies indicate the spasmolytic and anti-inflammatory properties of the constituents [11,12]. However, inflammatory crosstalk between immune cells and intestinal epithelial cells (IECs) as well as inflammation-driven mucosal barrier dysfunction are pivotal to the complex IBD pathogenesis [13,14]. To improve our understanding of the mode of action of the herbal combination, particularly in the field of IBD, the present study aimed to evaluate the effects of myrrh, coffee charcoal and chamomile flower extract on these two key characteristics. A complex co-culture cell model, featuring THP-1 macrophages and a monolayer of enterocyte-like Caco-2 cells, combined with goblet cell-like HT29-MTX-E12 cells, was established to allow the simultaneous investigation of the pro-inflammatory communication between immune and epithelial cells and the resulting IEC barrier disorders. Subsequently, the effects of myrrh, coffee charcoal, and chamomile flower extract as well as a combination of all three extracts compared to budesonide were evaluated via the quantification of inflammatory mediator secretion (TNF, IL-6, IL-8, MCP-1 and PGE_2_) into the supernatant and the measurement of the transepithelial electrical resistance of the IEC monolayers.

## 2. Materials and Methods

### 2.1. Chemicals

Ethanol for extraction was purchased in HSL quality from CSC Jäcklechemie (Rauschwitz, Germany) and purified by evaporation. Chemicals in p.a. grade were supplied by VWR (Radnor, PA, USA; heptane, n-hexane) or Thermo Fisher Scientific (Waltham, MA, USA; formic acid, ethyl acetate, methanol), whereas HPLC-grade methanol and acetonitrile were provided by Merck Chemicals (Darmstadt, Germany). Curzerenone and furanoeudesma-1,3-diene were purchased from PhytoLab(Vestenbergsgreuth, Germany). Chloroform-d_3_, phorbol-12-myristate-13-acetate (PMA), budesonide (bud), lipopolysaccharides from *Escherichia coli* O111:B4 (LPS), thiazolyl blue (MTT), Triton X-100 and valinomycin were obtained from Sigma Aldrich (St. Louis, MO, USA). The cell culture supplies DMEM High Glucose, RPMI-1640, fetal bovine serum (FBS), penicillin–streptomycin (P/S), glutamine stable, MEM non-essential amino acids (NEAA), trypsin–EDTA, and Dulbecco’s phosphate buffered saline (DPBS) were provided by Biowest (Riverside, MO, USA). The 2-Mercaptoethanol was obtained from Gibco (Life Technologies, Carlsbad, CA, USA), the isopropanol from Carl Roth (Karlsruhe, Germany) and the hydrochloric acid 1 mol/L (1 N) from Tritipur (Merck, Darmstadt, Germany).

### 2.2. Plant Extraction and Phytochemical Profiling

Powdered myrrh (Myrrhe Gum EB/BP pulv., batch-no. NM0160) and powdered coffee charcoal (*Carbo Coffea* EB 6, batch-no. JB0142) as source materials as well as ready-to-use chamomile flower dry extract (EtOH 60% *m*/*m*; DER: 4–6:1; batch-no. HC0070) were provided by Lomapharm (Emmerthal, Germany).

The coffee charcoal extract was prepared, lyophilized and stored as previously described by Weber et al. [15].

An ethanolic myrrh extract was obtained yielding 761.65 g (DER: 3.9:1) by alternating the percolation and maceration of 3 kg powdered myrrh for seven days with 26 L ethanol 96% (*v*/*v*). The solvent was removed with a rotary evaporator and the extract lyophilized and stored light-protected at −20 °C until further use.

Since the phytochemical composition of the chamomile flower [12] and coffee charcoal extract [15] have been described before, the phytochemical extract characterization in this study was limited to the myrrh extract. To gain further reference substances besides furanoeudesma-1,3-diene (**1**) and curzerenone (**2**), the dried myrrh extract was separated in a liquid–liquid partition between n-heptane and methanol. Subsequently, the n-heptane fraction was separated by silica flash chromatography (Spot flash system (Armen Instrument, Paris, France); column: SVP D40, SI60 15–40 µm, 90 g (Götec Labortechnik GmbH, Bickenbach, Germany); eluent A: hexane, eluent B: ethyl acetate; gradient: 0–30 min, 5% B; 30–90 min, 5–10% B; 90–110 min, 10–100% B; 110–120 min, 100% B; flow: 30 mL/min), centrifugal partition chromatography (Spot CPC system (Armen Instrument); eluent system: hexane/acetonitrile/methanol (8/5/2); stationary phase: lower phase; mobile phase: upper phase; flow: 5 mL/min) and preparative HPLC (Binary 1260 HPLC Infinity with 1260 manual injector, 1260 DAD and 1260 fraction collector (Agilent Technologies, Santa Clara, CA, USA); column: Biphenyl, 250 × 21.2 mm 5 µm, 100 Å (Agilent Technologies); eluent A: water; eluent B: acetonitrile; flow: 21 mL/min; different gradients were used). The primary structure elucidation of the isolated compounds was accomplished using 1D and 2D NMR analyses in chloroform-d_3_ (Avance III 600 NMR with 5 mm TCI CryoProbe; 600.25 MHz (^1^H-NMR) und 150.95 MHz (^13^C-NMR) (Bruker Corporation, Billerica, MA, USA)). Thus, by the comparison of the experimental data with the literature, all the components could be unequivocally identified by their chemical shifts, splitting patterns and coupling constants.

To verify the presence of previously isolated compounds in the extract, reference substances and myrrh extract were analysed with LC–MS (Q-TOF 6540 UHD, Agilent) on a ZORBAX Eclipse XDB-C18 RRHD, 2.1 × 100 mm, 1.8 µm (Agilent Technologies) with solvent A (water with 0.1% formic acid) and solvent B (acetonitrile with 0.1% formic acid): gradient: 0–10 min, 20–98% B; 10–12 min, 98% B; 12–12.1 min, 98–20% B; 12.1–13.5 min, 20% B with a flow of 0.5 mL/min, a column temperature of 50 °C and an injection volume of 1 µL. Subsequently, the MS analysis was performed with electrospray ionization (ESI) in positive and negative mode. The substances, which were not accessible by LC–ESI were measured with GC–MS (7890B GC (Agilent) and AccuTOF GCX (Jeol, Freising, Germany)) on a Zebron ZB-5MSplus column (Phenomenex, Aschaffenburg, Germany) and a helium flow of 1.2 mL/min. The temperature of the column oven was held at 80 °C for 0.5 min, then increased at a rate of 25 °C/min to 320 °C and was held for 5 min. Detection was performed by electron ionisation.

### 2.3. Cell Culture Model of the Intestinal Mucosa

THP-1 cells (ATCC (Manassas, VA, USA), TIB-202) served as a model for the macrophage function. The cells were cultured in RPMI-1640 supplemented with 10% FBS, 1% P/S and 0.1% 2-mercaptoethanol. Enterocyte-like Caco-2 cells (DMSZ (Braunschweig, Germany), ACC 169) and goblet cell-like HT29-MTX-E12 cells (ECACC (Public Health England, Salisbury, UK), 12040401) were used to model the intestinal epithelial cell (IEC) monolayer. The culture media consisted of DMEM supplemented with 10% FBS, 1% P/S, 1% NEAA and in the case of the HT29-MTX-E12 cells and IEC monolayers 1% glutamine stable. All the cell lines were maintained under standard cell culture conditions. To model the IEC monolayers, Caco-2 and HT29-MTX-E12 cells (ratio 9:1) were seeded in transwell inserts (33,000 cells per insert; Sarstedt (Nümbrecht, Germany), 0.4 µm pore size, PET) in a 24-well plate (BD Falcon (BD Biosciences, Billerica, MA, USA)) and differentiated for 21 days with a change of the culture media three times per week. The THP-1 cells were differentiated to macrophage-like cells in 24-well plates by treatment with PMA for 48 h (100,000 cells per well). After the differentiation period, the IEC monolayers and the THP-1 macrophages were combined to create the co-culture cell model of the intestinal mucosa.

### 2.4. Inflammatory Stimulation and Substance Incubation

For the inflammatory activation, the THP-1 macrophages were challenged with LPS (100 ng/mL). The resulting release of pro-inflammatory mediators led to the inflammatory stimulation of the IEC monolayer.

Concomitantly, various concentrations of the plant extracts (myrrh 0.1–100 µg/mL, coffee charcoal 1–500 µg/mL, chamomile flower 0.1–200 µg/mL, combination of equal parts of all three extracts 0.25–150 µg/mL) were added to the cell culture medium of IECs (apical compartment) and THP-1 macrophages (basolateral compartment). Budesonide 0.1 µM served as a positive control. The following untreated controls were employed: stimulated control (s), inactivated control (ua; without the LPS activation of macrophages), unstimulated control (us; IEC monolayers without macrophages).

### 2.5. Transepithelial Electrical Resistance (TEER) Measurement

The TEER of the IEC monolayers in Ohm was determined before and 48 h after the stimulation and substance incubation with an EVOM^2^ epithelial volt-ohmmeter plus a STX3 electrode (World Precision Instruments).

### 2.6. Mediator Protein Release Quantification

The cytokine (IL-6, TNF) release of the THP-1 macrophages (basolateral compartment) and the chemokine (IL-8, MCP-1) release of the IEC monolayers (apical compartment) was quantified in the cell-free supernatant using respective BD OptEIA™ ELISA Sets (BD Biosciences, Billerica, MA, USA) according to the manufacturer’s instructions after 4, 24 and 48 h. A competitive ELISA (Prostglandin E_2_ ELISA Kit –Monoclonal, Cayman Chemical, Ann Arbor, MI, USA) was used for the quantification of PGE_2_ release into the supernatant after 48 h, as described by the manufacturer.

### 2.7. Cell Viability Testing

#### 2.7.1. MTT Assay

In order to evaluate cell viability, the metabolic activity of the macrophages was tested with a subsequent MTT assay after 48 h treatment. After the removal of the supernatant, the cells were incubated with 500 µL MTT (thiazolyl blue) solution (0.3 mg/mL in DPBS) at 37 °C for 2 h. Resulting formazan salt was dissolved with 500 µL formazan solvent (0.04 M HCl in isopropanol + 0.1% Triton X-100) overnight. The formazan content was quantified spectrophotometrically at 570 nm with an Infinite^®^ 200 PRO plate reader (Tecan, Männedorf, Switzerland).

#### 2.7.2. LDH Assay

In addition, the cell-free IEC and macrophage supernatant was tested for extracellular lactate dehydrogenase (LDH) activity after 48 h using the Cytotoxicity Detection Kit^PLUS^ (LDH) provided by Roche (Basel, Switzerland) according to the manufacturer’s instructions.

#### 2.7.3. JC-10 Assay

Moreover, a JC-10 assay was conducted to test for the apoptosis of the IEC monolayers 48 h after treatment and stimulation. The Mitochondrial Membrane Potential Kit (Sigma Aldrich, St. Louis, MO, USA) was utilized in accordance with the manufacturer’s instructions. Valinomycin (val, 100 µg/mL) served as a positive control for apoptosis.

#### 2.7.4. BrdU Assay

The proliferation of IEC monolayers was assessed following 48 h stimulation and treatment with a BrdU assay (Cell Proliferation ELISA, BrdU (colorimetric), Roche, Basel, Switzerland) according to the manufacturer’s instructions.

### 2.8. Data Analysis

Microsoft Excel 2013 was used for data capturing and processing from the voice recordings of the TEER measurements and spectrophotometric data of the mediator release and viability experiments. The TEER difference was calculated by the subtraction of the TEER measured 48 h after stimulation and treatment from the initial value. 

For the statistical evaluation, an ordinary one-way (TEER, PGE_2_, viability assays) or two-way (cytokine/chemokine release) analysis of variance (ANOVA) followed by Dunett’s multiple comparisons test against a stimulated control was conducted with GraphPad Prism 8.0.2 (* *p* < 0.05, ** *p* < 0.01, *** *p* < 0.001, **** *p* < 0.001). The half-maximal inhibitory and effective concentration (IC50 and EC50) were computed based on the non-linear regression of the concentration–response curves. IC50 was calculated based on the absolute protein release values with the stimulated control as an upper constraint for non-linear regression. The percentage of mediator release was plotted by setting the stimulated control to equal 100%. The normalized TEER differences (stimulated control = 0%, unstimulated = 100%) were used to compute the EC50. 

To assess for the synergistic, additive or antagonistic interaction of the individual components in the combination of equal parts of all three extracts and potential dose-reducing effects, the combination index (CI) was calculated based on the methods developed by Chou and colleagues [16] with the following equation:

CI = (IC50_comb_/IC50_A_) + (IC50_comb_/IC50_B_) + (IC50_comb_/IC50_C_)

IC50_A_, IC50_B_ and IC50_C_ are the half-maximal inhibitory concentrations of the individual extracts of myrrh, coffee charcoal and chamomile flower, respectively. IC50_comb_ is equivalent to one third of the IC50 calculated for the effect of the combination of the equal parts of the three extracts. If a component had no significant effect in an assay, the corresponding term was extracted from the equation. The CI for the effects on TEER was obtained analogously with the EC50.

Based on the categorisation of Chou [16], an CI between 0.9 and 1.10 (0) indicates nearly additive effects. The CI values < 0.9 describe the synergistic interaction with slight synergism ranging from 0.85 to 0.90 (+), moderate synergism from 0.7 to 0.85 (++), synergism from 0.3 to 0.7 (+++) and strong synergism from 0.1 to 0.3 (++++). A CI > 1.10 describes an antagonistic interaction (1.10–1.20 slight antagonism (−), 1.20–1.45 describes moderate antagonism (−−), 1.45–3.3 describes antagonism (−−−), and 3.3–10 describes strong antagonism (−−−−)).

## 3. Results and Discussion

### 3.1. Phytochemical Profiling

**Figure 1:**
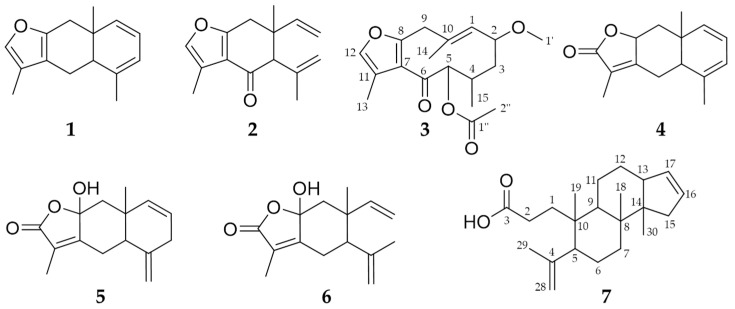
Chemical structures of the compounds identified in the myrrh extract: furanoeudesma-1,3-diene (**1**), curzerenone (**2**), 2-methoxy-5-acetoxyfuranogermacr-1(10)-en-6-one (**3**), 5-αH,8-βH-eudesma-1,3,7(11)-trien-8,12-olide (**4**), hydroxylindestrenolide (**5**), hydroxyisogermafurenolide (**6**) and 3,4-secomansumbinoic acid (**7**).

Phytochemical profiling was conducted to receive an impression of the extract composition, and identify a spectrum of substances, which might contribute to the effects observed in pharmacological testing, to ensure extract quality and allow for standardisation. Among others, the presence of seven characteristic compounds (Figure 1) in the myrrh extract was revealed through the comparison of retention times and MS data with authentic compounds (Table S1 and Figure S1). Besides two commercially available substances, furanoeudesma-1,3-diene (**1**) and curzerenone (**2**), five compounds were isolated and identified as 2-methoxy-5-acetoxyfuranogermacr-1(10)-en-6-one (**3**) [17], 5-αH,8-βH-eudesma-1,3,7(11)-trien-8,12-olide (**4**) [18], hydroxylindestrenolide (**5**) [19,20], hydroxyisogermafurenolide (**6**) [19,20] and 3,4-secomansumbinoic acid (**7**) [21,22] by comparing 1D, 2D NMR and MS data with the literature and used as references (Tables S2 and S3). 

The phytochemical composition of the other two extracts has been previously investigated and described. Caffeine, trigonelline, chlorogenic acid, neochlorogenic acid, cryptochlorogenic acid, feruloylquinic acid isomers and a caffeoylquinolactone were reported as compounds of the coffee charcoal extract [15], while the extract characterization of the chamomile flower extract indicated apigenin and its glycosides as its main components, accompanied by (iso) ferulic acid glycosides [12].

### 3.2. Pharmacological Evaluation of Plant Extracts with In Vitro Model of the Inflamed Intestinal Mucosa

In order to emulate an intestinal inflammation process with aberrant pro-inflammatory crosstalk between the intestinal epithelium and immune cells, as well as a disruption of the epithelial barrier function in vitro, a co-culture cell model of the intestinal mucosa was established.

**Figure 2:**
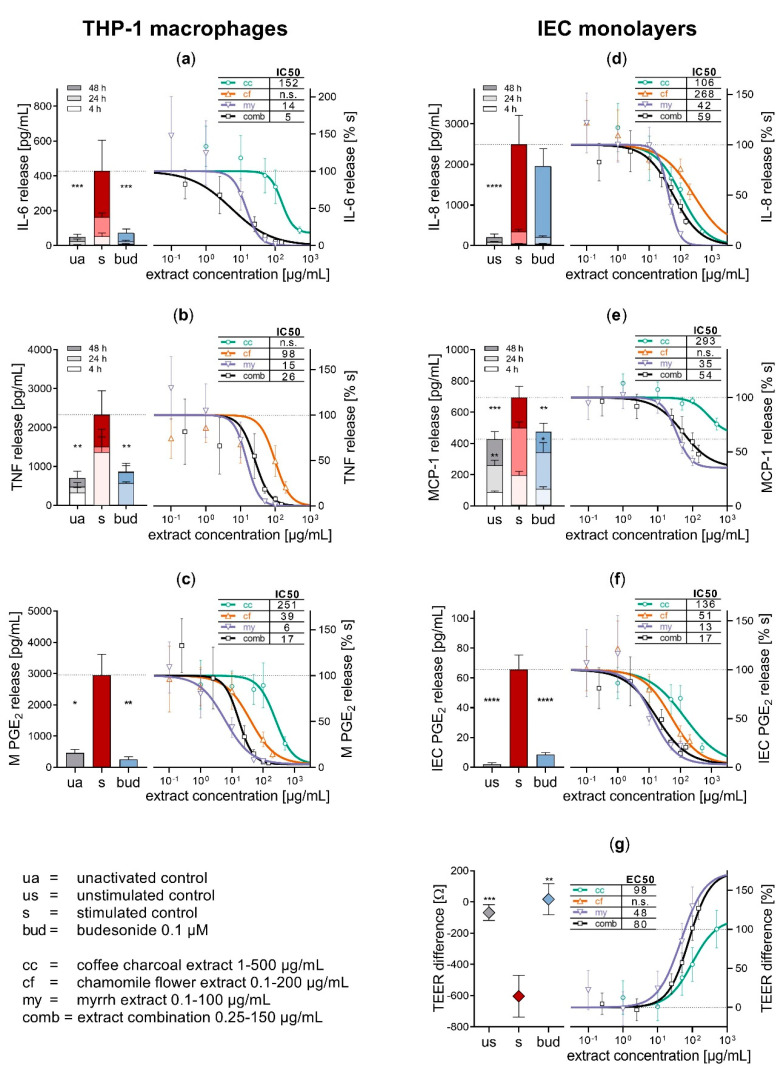
Pharmacological effects of myrrh, coffee charcoal and chamomile flower extract and the three-fold combination on inflammatory mediator release from the THP-1 macrophages (**a**–**c**) and the intestinal epithelial cell (IEC) monolayers (**d**–**f**) and transepithelial electrical resistance (TEER) difference (**g**) after 48 h of inflammatory stimulation and treatment in comparison to the controls. Mean ± standard error of the mean. * *p* < 0.05, ** *p* < 0.01, *** *p* < 0.001, **** *p* < 0.001.

Inflammation was induced by activating THP-1 macrophages with LPS (100 ng/mL, 48 h), which led to a significant increase in the pro-inflammatory cytokine release compared to inactivated cells (IL-6 8.5-fold, *p* < 0.001, Figure 2a; TNF 3.3-fold, *p* < 0.01, Figure 2b). This induction of cytokine secretion caused an inflammatory stimulation of the intestinal epithelial cell (IEC) monolayers, resulting in an enhanced chemokine release (IL-8 12.1-fold, *p* < 0.001, Figure 2d; MCP-1 1.6-fold, *p* < 0.001, Figure 2e; after 48 h). The release of PGE_2_, a representative of eicosanoid mediators, was increased in both macrophages (6.4-fold, *p* < 0.05; Figure 2c) and epithelial cells (34-fold, *p* < 0.01; Figure 2f) compared to the inactivated or unstimulated control, respectively.

As a result of the inflammatory stimulation, the barrier function of the IEC monolayers was diminished, reflected by a significantly lowered TEER difference after 48 h (*p* < 0.01, Figure 2g) compared to the unstimulated control. 

The topically active glucocorticoid budesonide was chosen as an experimental positive control because of its known inhibitory effects on cytokine and eicosanoid synthesis [23,24] and prominence in the treatment of inflammatory diseases like mildly to moderately active Crohn’s disease and asthma [24,25]. Budesonide 0.1 µM significantly reduced the release of cytokines from activated macrophages, and PGE_2_ from both macrophages and IECs (TNF and PGE_2_ *p* < 0.01, IL-6 *p* < 0.001). MCP-1 chemokine release from stimulated IECs was significantly reduced as well (*p* < 0.01), while no significant reduction of IL-8 secretion was observed.

In addition to anti-inflammatory activity, budesonide exerts an IEC barrier-stabilising effect, which might be caused by an increased expression of sealing and a down-regulation of pore-forming tight junctions described for glucocorticoids [26]. Concomitant treatment with budesonide 0.1 µM compensated the barrier disruptive effect of the inflammatory stimulation of the IEC monolayers (*p* < 0.01). Absolute mediator release and TEER difference of the controls is shown in Figure 2.

This co-culture model unites features of Caco-2/HT29-MTX-mucosa models that focus on intestinal epithelial cell function [27] with an experimental approach for inflammatory stimulation by involving THP-1 macrophages [28]. Thus, it allows to simultaneously investigate the influence of test substances on intestinal immune response via intercellular crosstalk, and barrier function. Compared to the in vivo microenvironment of the human intestine, this in vitro cell model lacks genetic heterogeneity and individual exposure present in patients, and has a limited complexity regarding cell types and the signalling pathways present. However, it provides a cost-effective approach for the pharmacological screening and mechanistic evaluation of phytotherapeutic strategies in IBD treatment with standardised and reproducible experimental conditions, suitable for extensive application. 

In order to further elucidate the mechanisms of action in IBD therapy, the herbal components of Myrrhinil-Intest^®^ were tested for anti-inflammatory and barrier-stabilising activity, individually and combined. The combination of equal parts of myrrh, coffee charcoal and chamomile flower extract reduced the inflammatory crosstalk between the immune cells and IECs, via the inhibition of the secretion of all examined inflammatory mediators. The individual extracts contributed to this effect to a varying extent, with myrrh showing the most distinct impact on both THP-1 macrophages and IECs. This observation is in line with previous studies on anti-inflammatory activity [12]. Figure 2a–f displays the concentration response curves for the inhibition of inflammatory mediator release by the individual extracts as well as the threefold combination. 

Myrrh and coffee charcoal extract as well as the combination of all three extracts exerted IEC barrier-stabilising effects through a concentration-dependent increase in the TEER difference after 48 h inflammatory stimulation (Figure 2g). These results confirm the previously reported intestinal barrier-enhancing effects for myrrh in a TNF-stimulated Caco-2 pure cell model [29] and to our knowledge, provide the first observation of a barrier-stabilising effect of coffee charcoal extract. This barrier-stabilising activity could be evoked by both the reduction of excessive inflammatory mediator release and a modification of tight junction proteins, as it was previously suggested for myrrh and glucocorticoids [26,29].

For the assessment of the pharmacological interaction between the individual extracts combination indices (CI) were calculated and categorised after Chou [16], and are summarized alongside the IC50 and EC50 values in Table 1. The results imply strong synergism for the inhibition of IL-6 release from macrophages, synergistic inhibition of TNF release from macrophages and MCP-1 as well as PGE_2_ release from the IECs, while interaction in the inhibition of PGE_2_ from the macrophages was slightly antagonistic. Moderate synergism occurred in the inhibition of IL-8 release from the IECs as well as with the TEER-enhancing effects of myrrh and coffee charcoal within the threefold combination. Thus, the ternary combination offers the potential to achieve certain effect levels with lower concentrations of the individual extracts.

To ensure cell integrity and assess the potential cytotoxicity, the cell viability and proliferation of epithelial cells was evaluated via LDH, JC-10 and BrdU assay (Figure S2). The THP-1 macrophage viability was assessed with the LDH and MTT assay (Figure S3). The LPS activation of the THP-1 macrophages and the inflammatory stimulation of the IECs as well as the treatment with budesonide, coffee charcoal and chamomile flower extract, did not limit the cell viability in the tested concentrations. The treatment with myrrh extract had no significant effects on cell viability, yet at a concentration of 100 µg/mL, it led to a reduction of the metabolic activity of macrophages by 65% and the mitochondrial membrane potential of IECs by 32% (*p* > 0.05), which points towards a cytotoxic potential of myrrh extract in higher concentrations.

## 4. Conclusions

The presented co-culture cell model of the intestinal mucosa provides an opportunity for the combined investigation of test substances regarding their effects on the intestinal immune response through cytokine/chemokine signaling and inflammation-promoted barrier dysfunction, two key factors in the pathophysiology of certain intestinal diseases such as IBD.

In this context, with the available clinical evidence suggesting the efficacy of the herbal combination of myrrh, coffee charcoal and chamomile flower extract in the maintenance therapy of Crohn’s disease, our study provides a pharmacological and mechanistic rationale for the use of the traditional herbal medicine for the treatment of inflammation-associated gastrointestinal disorders. The results underline that the phytotherapeutic multi-component/multi-target approach can be beneficial in the treatment of the complex pathogenesis of IBD by exerting many faceted effects and synergistic interaction.

## Figures and Tables

**Table 1:** Pharmacological interaction within the ternary combination of equal parts of myrrh, coffee charcoal and chamomile flower extract. Summary of the half-maximal inhibitory or effective concentrations (IC/EC50) and the respective combination indices for the effects on inflammatory mediator release from the THP-1 macrophages (M; IL-6, TNF, PGE_2_) and intestinal epithelial cells (IECs; IL-8, PGE_2_) and transepithelial electrical resistance (TEER) after 48 h stimulation and treatment. 95%CI = 95% confidence interval, (++++) = strong synergism, (+++) = synergism, (++) = moderate synergism, (−) = slight antagonism.

Mediator Release	Myrrh (0.1–100 µg/mL)	Coffee Charcoal (1–500 µg/mL)	Chamomile Flower (0.1–200 µg/mL)	Extract Combination (0.25–150 µg/mL)	
	IC50 (µg/mL) (95%CI)	IC50 (µg/mL) (95%CI)	IC50 (µg/mL) (95%CI)	IC50 (µg/mL) (95%CI)	Combination Index
M-IL-6	**14** (3.3–62.3)	**152** (2.1–10905)	-	**5** (1.9–15.6)	**0.13 (++++)**
M-TNF	**15** (5.1–44.5)	-	**98** (54.8–175.6)	**26** (13.7–49.7)	**0.67 (+++)**
M-PGE_2_	**6** (1.5–17.9)	**251** (105.2–3005)	**39** (7.9–116.4)	**17** (4.2–33.2)	**1.11 (**−**)**
IEC-IL-8	**42** (20.5–86.1)	**106** (46.1–244.9)	**268** (40.1–1790)	**59** (34.1–103.5)	**0.73 (++)**
IEC-MCP-1	**35** (9.9–122.5)	**293** (107.3–800.2)	-	**54** (27.1–106.7)	**0.58 (+++)**
IEC-PGE_2_	**13** (2.9–64.1)	**136** (51.8–532.0)	**51** (15.2–154.0)	**17** (4.5–39.6)	**0.59 (+++)**
**IEC barrier**	**EC50 (µg/mL)** **(95%CI)**	**EC50 (µg/mL)** **(95%CI)**	**EC50 (µg/mL)** **(95%CI)**	**EC50 (µg/mL)** **(95%CI)**	**Combination Index**
TEER	**48** (8.3–99.5)	**98** (15.2–3123)	-	**80** (35.7–102.0)	**0.83 (++)**

## Supplementary Materials

The following are available online at https://www.mdpi.com/2218-273X/10/7/1033/s1, Figure S1: Extracted ion chromatograms of myrrh extract in the GC–MS (A) and the LC–MS analysis in positive (B–E) and negative mode (F) observed for the expected masses of the seven reference substances with relevant peaks at their specific retention times (red), Table S1: Summary of LC–/GC–MS characteristics of the compounds identified in the myrrh extract, Table S2: 13C NMR spectroscopic data (150 MHz, CDCl3, δ in ppm) for compounds **3**–**7** isolated from the myrrh extract, Table S3: 1H NMR spectroscopic data (600 MHz, CDCl3, δ in ppm, J in Hz) data for the compounds **3**–**7** isolated from the myrrh extract (s singlet, d duplet, t triplet, br broad), Figure S2: Viability and proliferation of the IEC monolayers after 48 h of inflammatory stimulation and substance incubation assessed with JC-10- (**a**), LDH- (**b**) and the BrdU assay (**c**), Figure S3: Viability and proliferation of THP-1 macrophages after 48 h of LPS-activation and substance incubation assessed with MTT- (**a**) and LDH-assay (**b**).
