# Supplementary material for: Anti-Inflammatory and Barrier-Stabilising Effects of Myrrh, Coffee Charcoal and Chamomile Flower Extract in a Co-Culture Cell Model of the Intestinal Mucosa
Source: Biomolecules. 2020 Jul 11;10(7):1033. doi: 10.3390/biom10071033 (PMC7407830; doi:10.3390/biom10071033)
Supplement: Supplementary file 1 [file biomolecules-10-01033-s001.pdf]

biomolecules

Supplementary Material

Anti-Inflammatory and Barrier Stabilising Effects of Myrrh, Coffee Charcoal and Chamomile Flower Extract in a Co-Culture Cell Model of the Intestinal Mucosa

Laura Weber [1,2,*], Katrin Kuck [3], Guido Jürgenliemk [3], Jörg Heilmann [3], Bartosz Lipowicz [2] and Cica Vissiennon [1,2,*]

[1] Institute of Medical Physics and Biophysics, Medical Faculty, Leipzig University, Härtelstraße 16-18, 04107 Leipzig, Germany; laura.weber@medizin.uni-leipzig.de (L.W.), cica.vissiennon@uni-leipzig.de (C.V.);
[2] Repha GmbH Biologische Arzneimittel, Alt-Godshorn 87, 30855 Langenhagen, Germany; bartosz.lipowicz@repha.de (B.L.)
[3] Pharmaceutical Biology, Institute of Pharmacy, University of Regensburg, Universitätsstraße 31, 93053 Regensburg, Germany; katrin.kuck@chemie.uni-regensburg.de (K.K.), guido.juergenliemk@chemie.uni-regensburg.de (G.J.), joerg.heilmann@chemie.uni-regensburg.de (J.H.)
* Correspondence: laura.weber@medizin.uni-leipzig.de, Tel.: +49 341 97 15769 (L.W.); cica.vissiennon@uni-leipzig.de, Tel.: +49 341 97 15 708 (C.V.)

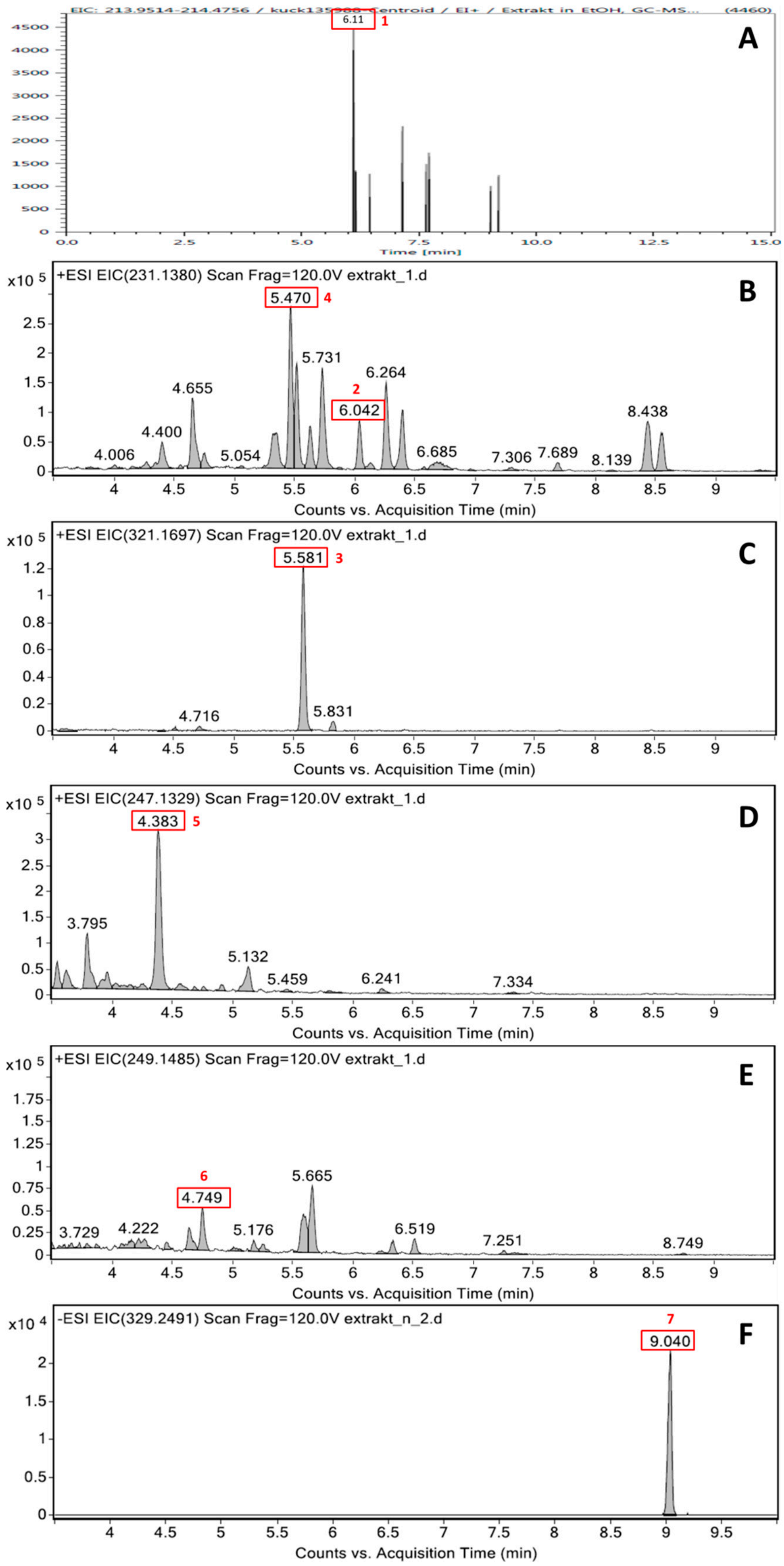

Figure S1. Extracted ion chromatograms of myrrh extract in GC-MS (**A**) and LC-MS analysis in positive (**B-E**) and negative mode (**F**) observed for the expected masses of the seven reference substances with relevant peaks at their specific retention times (red).

Table S1. Summary of LC-/GC-MS characteristics of compounds identified in myrrh extract. Retention times (RT in min) and mass spectra (m/z) of furanoeudesma-1,3-diene (**1**), curzerenone (**2**), 2-methoxy-5-acetoxyfuranogermacr-1(10)-en-6-one (**3**), 5-αH,8-βH-eudesma-1,3,7(11)-trien-8,12-olide (**4**), hydroxylindestrenolide (**5**), hydroxyisogermafurenolide (**6**) and 3,4-secomansumbinoic acid (**7**) determined in the standard solutions and the myrrh extract. Analyses were performed either on LC (a) or GC (b) in positive (c) or negative (d) mode (cf. section 2.3 of the article).

Standard	RT (standard)	*m/z* (standard)	RT (extract)	*m/z* (extract)	Molecular formula
1	6.11[a]	214.1345[c]	6.11[a]	214.1347[c]	$C_{15}H_{18}O$
2	6.045[b]	231.1281[c]	6.042[b]	231.1383[c]	$C_{15}H_{18}O_2$
3	5.587[b]	321.1700[c]	5.581[b]	321.1699[c]	$C_{18}H_{24}O_5$
4	5.473[b]	231.1379[c]	5.470[b]	231.1383[c]	$C_{15}H_{18}O_2$
5	4.402[b]	247.1330[c]	4.383[b]	247.1334[c]	$C_{15}H_{18}O_3$
6	4.761[b]	249.1487[c]	4.749[b]	249.1486[c]	$C_{15}H_{20}O_3$
7	9.024[b]	329.2491[d]	9.040[b]	329.2487[d]	$C_{22}H_{24}O_2$

Table S2. ^{13}C NMR spectroscopic data (150 MHz, $CDCl_3$, *δ* in ppm) for compounds 3-7 isolated from myrrh extract.

Pos.	3	4	5	6	7
1	132.8	136.0	137.5	147.4	34.3
2	73.8	122.8	123.1	112.0	28.2
3	37.8	120.2	34.8	114.2	179.8
4	30.6	136.7	145.3	144.8	147.5
5	78.9	46.7	49.9	54.2	51.0
6	195.8	23.7	24.0	27.1	24.7
7	121.2	163.8	160.8	160.3	34.1
8	154.3	78.8	103.,5	103.0	39.6
9	38.1	44.3	48.4	49.3	41.4
10	135.2	35.8	37.6	40.6	39.3
11	123.2	119.7	122.4	122.0	22.4
12	138.0	175.7	172.2	172.2	23.8
13	8.7	6.7	8.3	8.2	47.7
14	18.8	13.7	20.4	17.7	53.4
15	17.3	18.7	107.5	24.4	40.0
1′	55.8				
1′′	170.3				
2′′	20.7				
16					130.0
17					134.0
18					17.9
19					20.0
28					113.5
29					23.2
30					17.0

Table S3. ^{1}H NMR spectroscopic data (600 MHz, $CDCl_3$, δ in ppm, J in Hz) data for compounds 3-7 isolated from myrrh extract (s singlet, d duplet, t triplet, br broad).

Pos.	3	4	5	6	7
1	5.23 (1H, d, 9.9)	5.54 (1H, d, 9.4)	5.55 (1H, brd)	5.70 (1H, dd,<br>10.8/17.4)	1.62 (2H, m)
2	4.13 (1H, m)	5.79 (1H,<br>dd, 5.1/9.4)	5.48 (1H, ddd,<br>3.2/3.2/9.8)	4.97 (1H, brd, 17.4)<br>4.99 (1H, brd, 10.8)	2.21 (1H, dt, 8.9/16.1)<br>2.41 (1H, m)
3	1.,90 (1H, m)<br>1.90 (1H, m)	5.74 (1H, m)	2.79 (1H, brd, 20.4)<br>2.90 (1H, brd, 20.4)	4.73 (1H, brs)<br>4.98 (1H, brs)	
4	2.38 (1H, m)				
5	5.52 (1H, d, 8.4)	2.33 (1H,m)	2.14 (1H,brd, 13.1)	2.04 (1H, dd,<br>3.5/13.5)	2.00 (1H, dd,<br>3.8/11.9)
6		2.45 (1H,<br>dd, 13.8/13.8)<br>3.08 (1H,<br>dd, 3.9/13.8)	2.52 (1H, dd,<br>13.1/13.1)<br>2.71 (1H, dd,<br>3.0 / 13.3)	2.55 (1H, dd,<br>3.5/13.5)<br>2.72 (1H, dd,<br>13.5/13.5)	1.39 (1H, m)<br>1.87 (1H, ddd,<br>3.3/12.9/25.9)
					1.25 (1H, dt, 3.2/12.9)<br>1.62 (1H, m)
8		4.99 (1H,<br>dd, 6.3/11.6)			
9	3.31 (1H, d, 16.8)<br>3.64 (1H, d, 16.8)	1.31 (1H,<br>dd, 11.6/11.6)<br>2.32 (1H,<br>dd, 6.3/11.6)	1.68 (1H, d, 13.6)<br>2.33 (1H, d, 13.6)	1.74 (1H, d, 14.0)<br>2.14 (1H, d, 14.0)	1.64 (1H, m)
11					1.34 (1H, m)<br>1.49 (1H, m)
12	7.03 (1H, s)				1.45 (2H, m)<br>1.74 (1H, m)
13	1.92 (3H, s)	1.81 (3H, s)	1.82 (3H, brs)	1.82 (3H, brs)	2.72 (1H, m)
14	1.95 (3H, s)	1.02 (3H, s)	1.09 (3H, s)	1.26 (3H, s)	
15	1.08 (3H, d, 7.1)	1.89 (3H, s)	4.76 (1H, brs)<br>4.98 (1H, brs)	1.77 (3H, s)	1.70 (1H, m)<br>2.35 (1H, brd)
1′	3.25 (3H, s)				
2′′	2.04 (3H, s)				
16					5.65 (1H, m)
17					5.56 (1H, m)
18					1.07 (3H, s)
19					0.87 (3H, s)
28					4.68 (1H, d, 1.5)<br>4.86 (3H, brs)
29					1.74 (3H, m)
30					1.01 (3H, m)

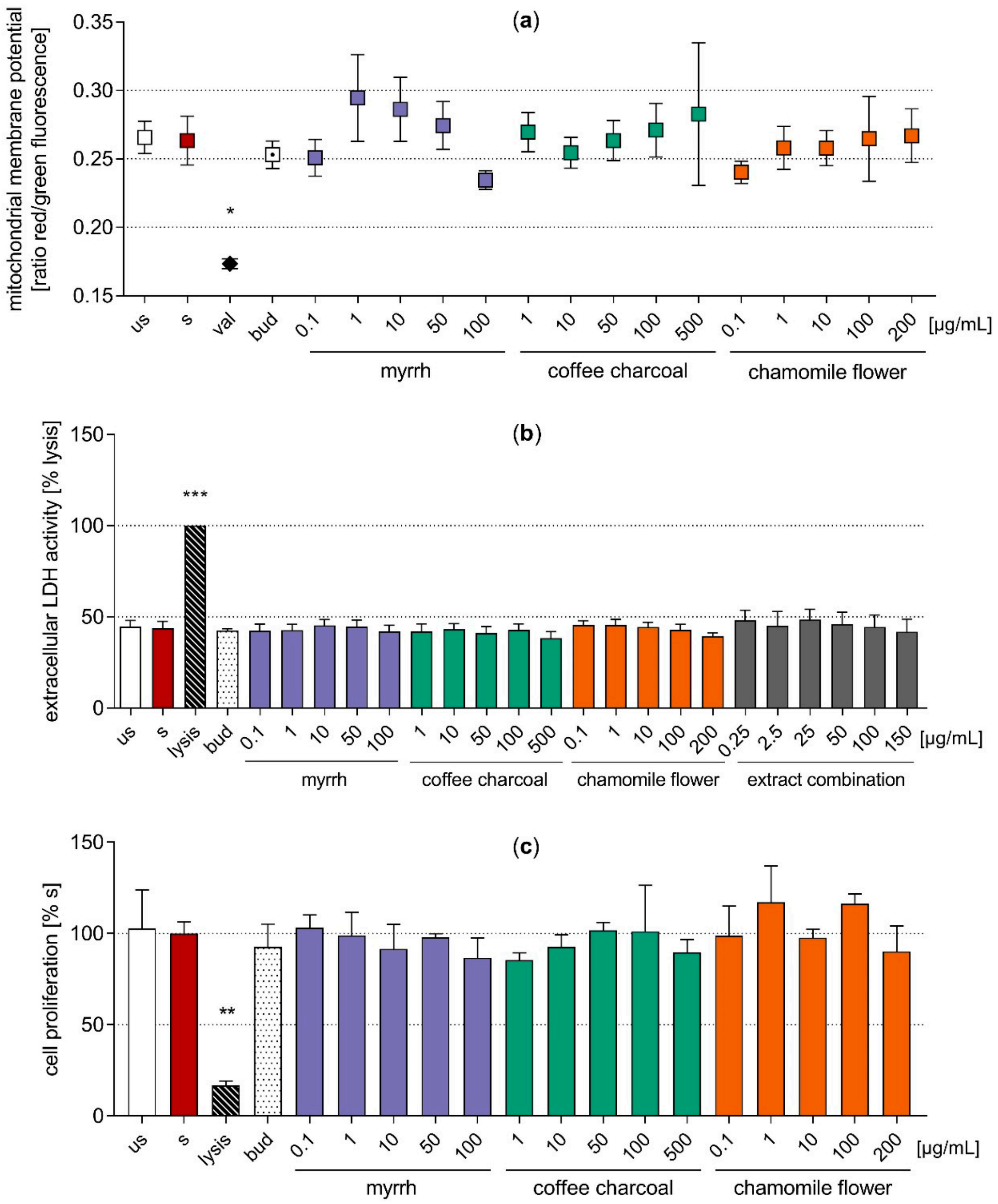

Figure S2. Viability and proliferation of IEC monolayers after 48 h of inflammatory stimulation and substance incubation assessed with JC-10- (**a**), LDH- (**b**) and BrdU-assay (**c**). us = unstimulated control, s = stimulated control, bud = budesonide 0.1 µM, val = valinomycin 100 µg/mL. Mean ± standard error of the mean, * = $p < 0.05$, ** = $p < 0.01$, *** = $p < 0.001$ in an ordinary one-way ANOVA followed by Dunnett's multiple comparisons test compared to the stimulated control.

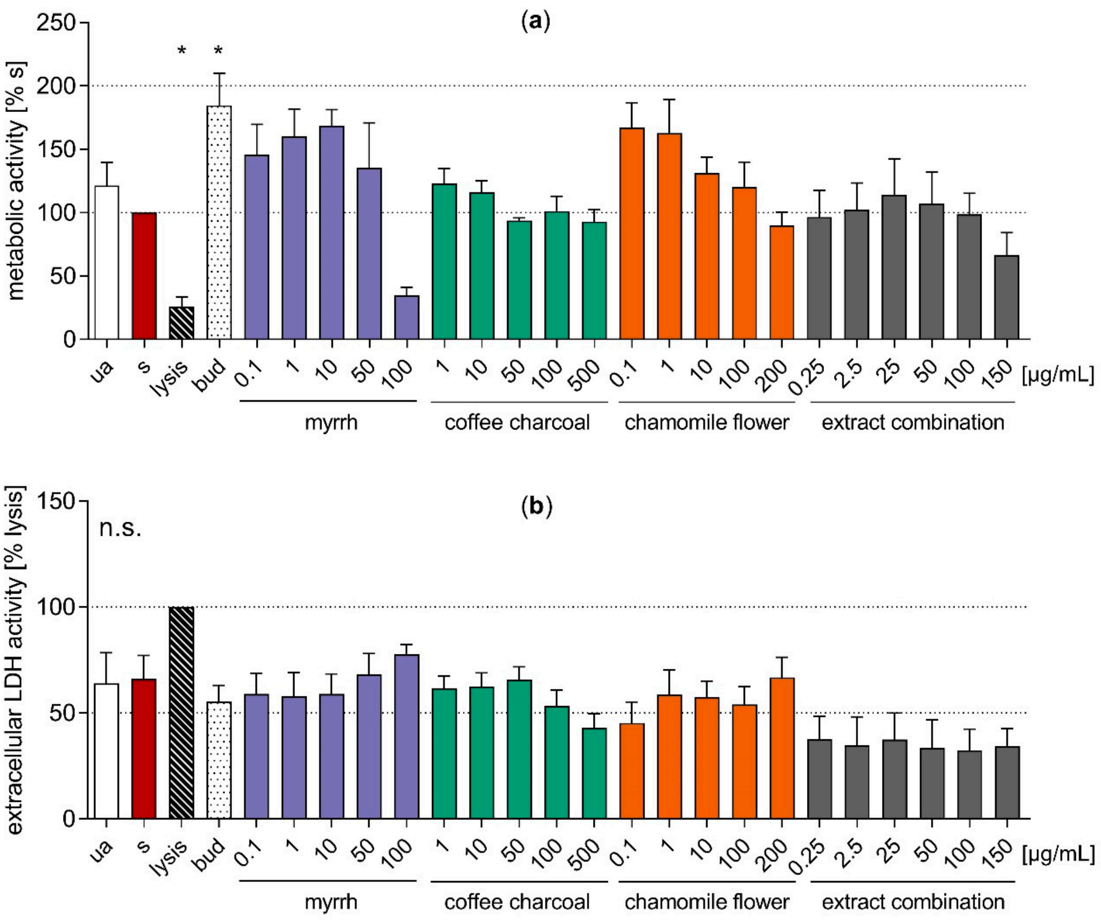

Figure S3. Viability and proliferation of THP-1 macrophages after 48 h of LPS-activation and substance incubation assessed with MTT- (**a**) and LDH-assay (**b**). ua = unactivated control, s = stimulated control, bud = budesonide 0.1 µM. Mean ± standard error of the mean, * = $p < 0.05$, ** = $p < 0.01$, *** = $p < 0.001$ in an ordinary one-way ANOVA followed by Dunnett's multiple comparisons test compared to the stimulated control.
